# The complete chloroplast genome sequence of *Ulmus lanceifolia* Roxburgh ex Wallich (Ulmaceae)

**DOI:** 10.1080/23802359.2021.2008845

**Published:** 2022-01-27

**Authors:** Lili Tong, Xiaoyu Jiang, Lu Tian, Xiaogang Xu, Chongli Xia, Yao Cheng, Hongchao Wang, Yukun Tian

**Affiliations:** a Co-Innovation Center for Sustainable Forestry in Southern China, College of Biology and the Environment, Key Laboratory of State Forestry and Grassland Administration on Subtropical Forest Biodiversity Conservation, Nanjing Forestry University, Nanjing, China; b State Environmental Protection Scientific Observation and Research Station for Ecology and Environment of Wuyi Mountains, Nanping, China; c School of Horticulture & Landscape Architecture, Jinling Institute of Technology, Nanjing, China

**Keywords:** *Ulmus lanceifolia*, complete chloroplast genome, Phylogenomics, Ulmaceae

## Abstract

*Ulmus lanceifolia*, which plays an important role in ecology and economy, is one of the main evergreen broad leaved arbor species in southern China. In this study, we assembled its complete chloroplast genome. The total genome size of *U*. *lanceifolia* was 158,652bp, containing a large single-copy region of 87,119bp, a small single-copy region of 18,697bp, and a pair of inverted repeat regions of 26,418bp. The overall GC content of *U*. *lanceifolia* chloroplast genome is 35.63%. We obtained the 132 genes chloroplast genome, including 86 proteincoding genes, 8 rRNA genes, and 37 tRNA genes. The phylogenetic analysis suggests that *U*. *lanceifolia* is a monophyletic taxa and a transitional species between the 2 groups which were combined by *Ulmus americana, U*. *elongate* and *Zelkova serrate*, *Z*. *schneideriana* respectively in Ulmaceae.

*Ulmus lanceifolia* (Roxburgh ex Wallich 1831) is one of the main evergreen broad leaved arbor species in southern China, with fairly good material toughness. It is around 30 meters tall, with a diameter of 40–80 centimeters at breast hight. Bark yellowish gray to chestnut brown, exfoliating in irregular flakes (Fu et al. 2003). The timber of *U*. *lanceifolia* is heavy and hard, not only is a good material for furniture, indoor decoration and plywood, but also suitable for vehicles, ships, farm tools, sporting goods and musical instruments (Xiao and Mu 1998). In addition, *U*. *lanceifolia* also has great potential in medicinal application. But to date, there is still no complete chloroplast (cp) genome is characterized for *U*. *lanceifolia.* The object of this work was to explore the intrinsic distinction in an effort to vindicate its taxonomic status in Ulmaceae. Here we present the cp genome of *U*. *lanceifolia* (GeneBank accession number: MZ159247) as an initial approach toward developing genomic tools to better understand *U*. *lanceifolia* and its close relatives.

The fresh leaves of *U*. *lanceifolia* were sampled from Xishuangbanna Tropical Botanical Garden (**latitude**101.262951, **longitude**21.931604), Yunnan, China. A specimen was deposited at the herbarium of Nanjing Forestry University (contact person: xuehongma@njfu.edu.cn) under the voucher number NF2021039. The whole genome sequencing was conducted using the Illumina NovaSeq platform by Nanjing Genepioneer Biotechnology Inc. (Nanjing, China). The raw reading was filtered by software fastp version 0.20.0, and the clean reading was used to assemble the chloroplast genome by SPAdes v3.10.1 (Bankevich et al. 2012). Finally, the gene structure was annotated using CpGAVAS2(Liu et al. 2012). The 20 complete chloroplast sequences were aligned by the MAFFT v7.475 (Katoh et al. 2019). Then we generated a maximum-likelihood (ML) tree through IQ TREE (Nguyen et al. 2015) with 1000 bootstrap alignments.

The complete chloroplast genome of *U*. *lanceifolia* was determined to be 158,652bp in length and contained 2 inverted repeat (IRa and IRb) regions of 26,418bp. The repeat regions divided the genome into 2 single-copy regions, SSC and LSC with 18,697bp and 87,119bp. A total of 132 genes are encoded, including 86 protein-coding genes, 37 tRNA genes, and 8 rRNA genes. Most of genes occurred in a single copy; however, 7 protein-coding genes (*ndhB*, *rpl2*, *rpl23*, *rps12*, *rps7*, *ycf15* and *ycf2*), 7 tRNA genes (*trnA*-*UGC*, *trnI*-*CAU*, *trnI*-*GAU*, *trnL*-*CAA*, *trnN*-*GUU*, *trnR*-*ACG* and *trnV*-*GAC*) and 4 distinct rRNA gene (*23S*, *16S*, *5S* and *4.5S*) are duplicated. A total of 18 protein-coding genes (*atpF*, *ndhA*, *ndhB*, *petB*, *petD*, *rpl16*, *rpl2*, *rpoC1*, *rps12*, *rps16*, *trnA*-*UGC*, *trnG*-*UCC*, *trnI*-*GAU*, *trnK*-*UUU*, *trnL*-*UAA*, *trnV*-*UAC*) contained 1 intron while the other 2 genes (*clpP*, *ycf3*) had 2 intron each. The overall GC content of *U*. *lanceifolia* genome was 35.63%, and the corresponding values in LSC, SSC and IR regions were 33.14%, 28.34%, and 42.33%, respectively.

The ML analysis reveals that *U*. *lanceifolia* is a monophyletic taxa and a transitional species between the 2 groups which were combined by *Ulmus americana, U*. *elongate* and *Zelkova serrate*, *Z*. *schneideriana* respectively in Ulmaceae (Figure 1).

**Figure 1.**
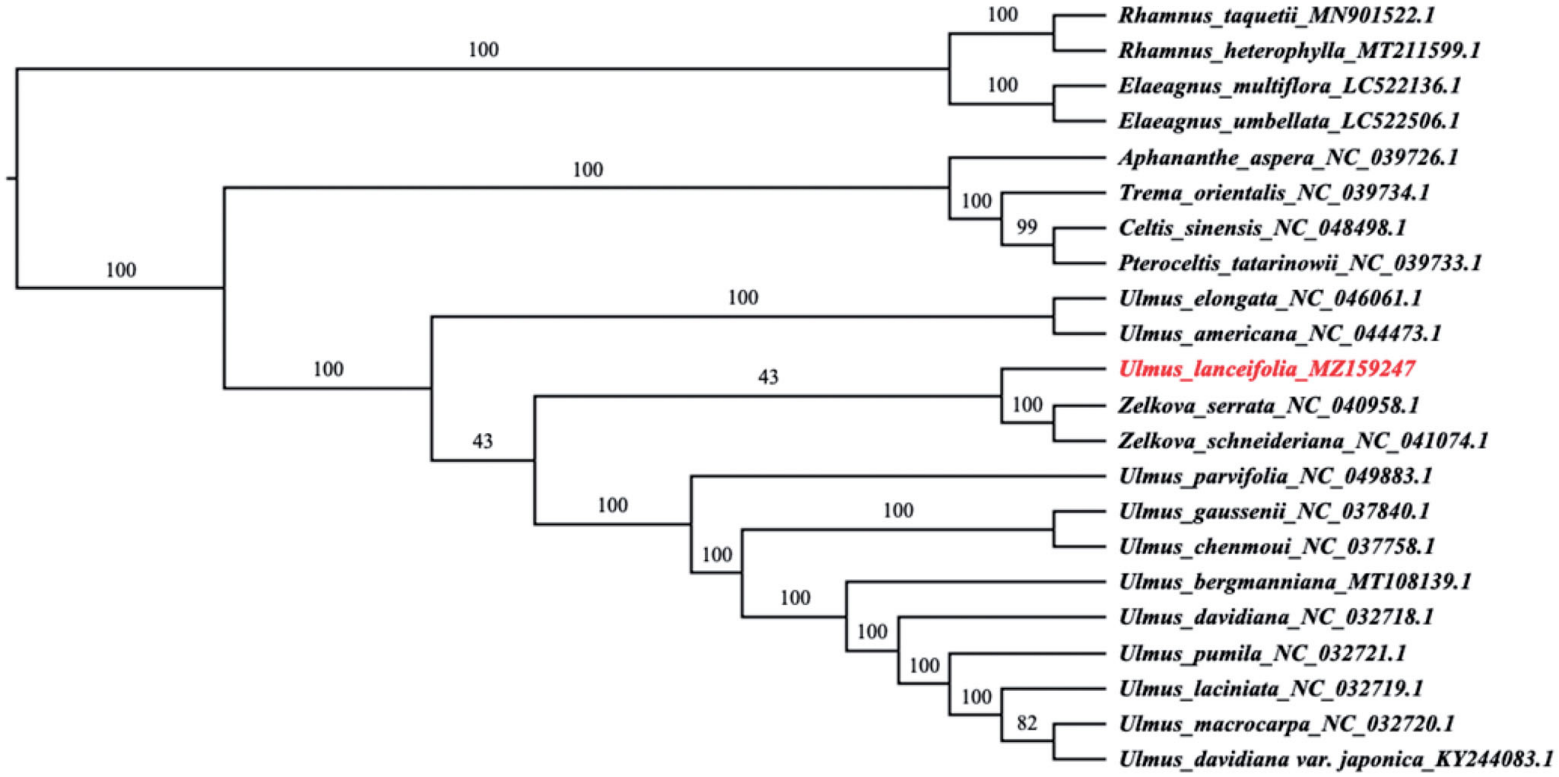
Phylogenetic relationships among 22 complete chloroplast genomes of Ulmaceae. *Elaeagnus multiflora*, *Elaeagnus umbellata*, *Rhamnus taquetii* and *Rhamnus heterophylla* were used as outgroups. The bootstrap supported the values shown at the branches.

## Data Availability

The data that support the findings of this study are openly available in the Genbank database at https://www.ncbi.nlm.nih.gov/, under accession number [MZ159247]. The associated BioProject, SRA, and Bio-Sample numbers are PRJNA753672, SRR15412756, and SAMN20695145 respectively.
